# *APOE* ε4 alters associations between docosahexaenoic acid and preclinical markers of Alzheimer’s disease

**DOI:** 10.1093/braincomms/fcab085

**Published:** 2021-05-11

**Authors:** Gillian Coughlan, Ryan Larsen, Min Kim, David White, Rachel Gillings, Michael Irvine, Andrew Scholey, Neal Cohen, Cristina Legido-Quigley, Michael Hornberger, Anne-Marie Minihane

**Affiliations:** 1 Norwich Medical School, University of East Anglia, Norwich, UK; 2 Rotman Research Institute, Baycrest, Toronto, ON, Canada; 3 Decision Neuroscience Laboratory, Beckman Institute for Advanced Science and Technology, University of Illinois, USA; 4 King’s College London, Franklin-Wilkins Building, London, UK; 5 Centre for Human Psychopharmacology, Swinburne University, Australia

**Keywords:** APOE genotype, docosahexaenoic acid, spatial navigation, entorhinal cortex, hippocampus

## Abstract

Docosahexaenoic acid is the main long-chain omega-3 polyunsaturated fatty acids in the brain and accounts for 30−40% of fatty acids in the grey matter of the human cortex. Although the influence of docosahexaenoic acid on memory function is widely researched, its association with brain volumes is under investigated and its association with spatial navigation is virtually unknown. This is despite the fact that spatial navigation deficits are a new cognitive fingerprint for symptomatic and asymptomatic Alzheimer’s disease. We investigated the cross-sectional relationship between docosahexaenoic acid levels and the major structural and cognitive markers of preclinical Alzheimer’s disease, namely hippocampal volume, entorhinal volume and spatial navigation ability. Fifty-three cognitively normal adults underwent volumetric magnetic resonance imaging, measurements of serum docosahexaenoic acid (DHA, including lysophosphatidylcholine DHA) and *APOE* ε4 genotyping. Relative regional brain volumes were calculated and linear regression models were fitted to examine DHA associations with brain volume. *APOE* genotype modulated serum DHA associations with entorhinal cortex volume and hippocampal volume. Linear models showed that greater serum DHA was associated with increased entorhinal cortex volume, but not hippocampal volume, in non *APOΕ* ε4 carriers. *APOE* also interacted with serum lysophosphatidylcholine DHA to predict hippocampal volume. After testing interactions between DHA and *APOE* on brain volume, we investigated whether DHA and *APOE* interact to predict spatial navigation performance on a novel virtual reality diagnostic test for Alzheimer’s disease in an independent population of *APOE* genotyped adults (*n* = 46). *APOE* genotype modulated DHA associations with spatial navigation performance, showing that DHA was inversely associated with path integration in *APOE* ε4 carriers only. This exploratory analysis suggests that interventions aiming to increase DHA blood levels to protect against cognitive decline should consider *APOE* ε4 carrier status. Future work should focus on replicating our initial findings and establishing whether a specific dose of supplementary DHA, at a particular time in the preclinical disease course can have a positive impact on Alzheimer’s disease progression in *APOE* ε4 carriers.

## Teaser

Docosahexaenoic acid’s neuroprotective effect may be concentrated in the entorhinal cortex and hippocampus. Here, we show that docosahexaenoic acid’s association with entorhinal volume and spatial navigation ability is modulated by the *APOE* genotype, with non-significant or inverse associations found in *APOE* ε4 carriers who are genetically-at-risk of Alzheimer’s disease.

## Graphical Abstract

**Graphical Abstract:**
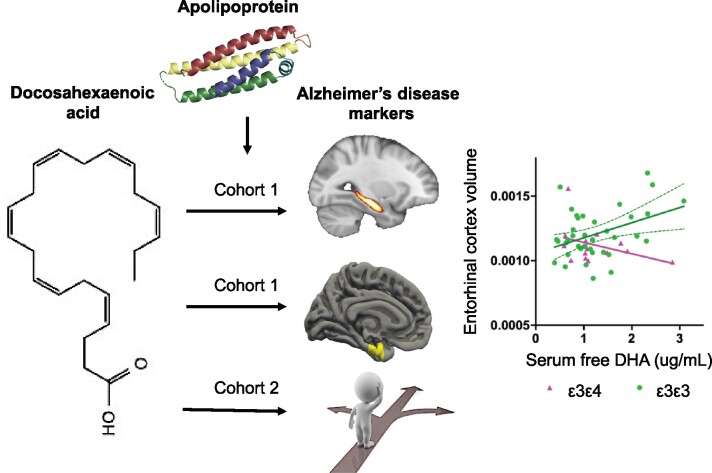

## Introduction

Alzheimer’s disease is the most common form of dementia with increasing world-wide prevalence. In the absence of any licensed drugs to treat or reverse cognitive decline associated with Alzheimer’s disease, dietary behaviours that prevent or slow brain atrophy in the entorhinal cortex and the hippocampus hold tremendous potential.^1–3^ Higher long-chain omega-3 polyunsaturated fatty acids (LC ω-3 PUFA) have been linked to better memory function and a lower risk of developing Alzheimer’s disease.^4–7^ However, the effect of LC ω-3 PUFA on spatial navigation is virtually unknown, despite evidence that spatial disorientation may appear in conjunction with, or prior to, episodic memory loss in Alzheimer’s disease.^8–10^ Therefore, elucidating the effect of LC ω-3 PUFA on spatial navigation and on its associated brain regions is of high interest.

Docosahexaenoic acid (DHA) is the main ω-3 PUFA in the brain and accounts for 30−40% of fatty acids in the grey matter of the human cortex. It is especially enriched at the synapse.^11^ Disturbances in brain DHA metabolism have been implicated in a host of neurodegenerative diseases, particularly Alzheimer’s disease. This may be because the beneficial effects of DHA appear to be concentrated in the hippocampus^12^ (particularly the CA1 subfield^6^) and the entorhinal cortex.^7^^,^^13^ Major animal studies show long-term DHA supplementation in mice with preclinical Alzheimer’s disease reverses amyloid accumulation, protects against neuronal loss associated with Alzheimer’s disease pathology, and critically, improves overall navigation performance.^6^^,^^14^ Conversely, reduced ω-3 PUFA levels has been shown to impair hippocampal plasticity and reduce navigation function,^15^^,^^16^ suggesting that DHA may be beneficial in prevention Alzheimer’s disease.

Despite brain DHA levels being 10-fold higher in specific brain regions relative to most body tissues, and strong evidence for neurocognitive benefits proposed by animal models, the neuronal benefits in humans are less consistent. In the Framingham Heart Study, higher serum phosphatidylcholine DHA levels were associated with a 47% reduction in risk of dementia over 9 years of follow-up,^17^ but in a similar dementia-free Dutch cohort, dietary DHA intake was not associated with relative risk for Alzheimer’s disease.^18^ Moreover, the Alzheimer’s Disease Cooperative Study reported no effect of DHA supplementation (18 months) on composite measures of cognition in adults with mild to moderate Alzheimer’s disease,^19^ but a subset of the Ageing Brain Study led by the University of Southern California shows higher serum DHA levels are associated with lower cerebral amyloidosis and preservation of entorhinal and hippocampal volumes.^7^

The unexamined role of the apolipoprotein (*APOE*) genotype may be a major factor behind the mixed results from human studies.^20–22^ The *APOE* ε4 isoform is the most important prevalent genetic determinant of Alzheimer’s disease risk^23^ and disrupts blood−brain barrier function in the hippocampus and wider medial temporal lobe, compared with the other *APOE* isoforms (ε2/ε3).^24^ This then suggests that a faulty blood−brain barrier system in *APOE* ε4 carriers may impair the transport of circulating DHA to the brain, which has been shown in older APOE ε4 mice.^25^ We hypothesized that the *APOE* genotype would modulate circulating blood DHA associations with select brain volumes and spatial navigation. We further hypothesized that higher DHA levels would be positively related to preserved brain entorhinal and hippocampal volume, as well as spatial navigation performance in non ε4 carriers only. We expected that these associations would be non-significant or negative in ε4 carriers.

## Materials and methods

This is a cross-sectional study examining the effect of *APOE* ε4 on DHA associations with entorhinal cortex volume, hippocampal volume and spatial navigation performance across two non-demented cohorts.

### Setting

Non-demented adults were drawn from the Cognitive Ageing, Nutrition and Neurogenesis study and formed Cohort 1.^26^ Recruitment and screening began in 2015 and all neuroimaging data were collected by March 2017 across two data collection sites; the Swinburne University of Technology (Melbourne, Australia) and the University of East Anglia (UEA, Norwich, UK). At screening, cognitive status was pre-classified with a modified telephone interview for cognitive status and Montreal Cognitive Assessment tool.^27^ Participants were invited for baseline neuropsychological testing and a baseline MRI scan. Blood samples were taken immediately following cognitive testing.

To investigate DHA associations with spatial navigation, we recruited a second cohort. Between February 2017 and June 2017, participants from this cohort were recruited to participate in a research study at Norwich Medical School, UEA and invited for spatial navigation and neuropsychological testing. Blood samples were taken immediately following testing.

### Standard protocol approvals, registrations and consents

The Cognitive Ageing, Nutrition and Neurogenesis study (ClinicalTrials.gov NCT02525198) obtained ethical approval from the Swinburne University Human Research Ethics Committee (Study identifier SHR Project 2015-208) for the Swinburne University of Technology site and the National Research Ethics Service Committee for the University of East Anglia site (Study identifier 14/EE/0189). Ethical approval for the second navigation study (Cohort 2) was obtained from the Faculty of Medicine and Health Sciences Ethics Committee at UEA, UK (Reference FMH/2016/2017-11). All participants from both studies provided informed signed consent before participating.

### Participants

Participants from Cohort 1 had a mean age of 64.7 (SD 7.6) years (*n* = 53). Participants from Cohort 2 were 61.3 (SD 5.6) years (*n* = 46). Exclusion criteria for both samples included diagnosis of mild cognitive impairment, clinical dementia, significant neurological/psychiatric disorder, MRI evidence of brain damage, previous vascular disorders including infarction or stroke and history of alcohol or drug dependency within the last 2 years. In addition, homozygous *APOE* ε4 carriers (2% of the population) and *APOE* ε2 carriers (13% of the UK population) were excluded, due to their low population prevalence. Included were (i) *APOE* ε3ε4 allele carriers, who are at a 3-fold increased risk of developing Alzheimer’s disease and represent 23% of the population (moderate risk, high prevalence) and (ii) age−gender matched ε3ε3 carriers, who represent the population wild-type genotype (60% of the population).^28^ Cohort 1 consisted of more ε3ε3 than ε3ε4 participants, while Cohort 2 consisted of almost equal numbers of ε3ε3 and ε3ε4 participants. This discrepancy between the studies was due to the original recruitment strategy (for further details, see Irvine et al.^26^ for Cohort 1 and Coughlan et al.^29^ for Cohort 2). All participants had normal or corrected-to-normal vision..

### *APOE* genotyping

In both cohorts, DNA was extracted and used for APOE genotyping. In Cohort 1, DNA was extracted from the buffy coat layer (containing the white cell layer) of each participants blood sample, and placed in a ethylenediaminetetraacetic acid tube (BD Biosciences, San Diego, CA, USA). In Cohort 2, DNA was extracted from a Darcon tip buccal swab (LE11 5RG, Fisher Scientific), using a commercial DNA extraction kit (Qiagen, Hildenberg, Germany). DNA from both samples underwent PCR amplification and plate read analysis using Applied Biosystems 7500 Fast Real-Time PCR System (TN23 4FD; Thermo Fisher Scientific) to determine participants’ *APOE* genotype status. Further information on the *APOE* genotyping procedure for each cohort is detailed elsewhere.^26^^,^^29^

### DHA measurements

#### Cohort 1

Free/non-esterified fatty acid serum DHA and lysophosphatidylcholine (LPC) serum DHA were measured from a fasted blood samples, with 20 µl of serum used for the analysis. Ten microlitres of high purity water and 40 µl of MS-grade methanol were added, followed by a 2 minute vortex mix to precipitate proteins. Two hundred microlitres of methyl *t*-butyl ether were added, and the samples were mixed via vortex at room temperature for 1 h. After the addition of 50 µl of high purity water, a final sample mixing was performed before centrifugation at 210 000 g for 10 min. The upper, lipid-containing, methyl *t*-butyl ether phase was then extracted and analysed by liquid chromatography−mass spectrometry. The analytical method is detailed elsewhere.^30^ The single molecule integrated peak areas under the exact mass chromatographs of LPC−DHA were obtained by using Skyline by setting up an integration parameter file using its mass charge ration (*m*/*z*) and retention time (510.35 *m*/*z* and 3.0 min).^31^^,^^32^ LPC DHA is the most important lipid pool to deliver DHA to the brain via the blood–brain barrier.^33–35^

#### Cohort 2

Erythrocyte DHA was measured from a non-fasted blood samples collected using a single drop of whole blood obtained via a finger prick collection kit (Faculty of Natural Sciences Institute of Aquaculture, University of Stirling). Blood samples were immobilized on a specially made card and sent to the University of Stirling (Stirling, UK) for analysis. See Carboni et al.^36^ for a full description of the Blood Spot PUFA analysis used to derive fatty acid erythrocyte concentrations. In Cohort 2, fasting status was not needed as DHA was measured in the erythrocyte phospholipid fraction, which is reflective or long-term (up to 3 months) fatty acid intake and little influenced by recent DHA or overall fatty acid intake.

### Volumetric MRI

Structural T_1_-weighted images were obtained using either a three-dimensional fast spoiled gradient echo brain volume imaging sequence in the sagittal orientation, repetition time (TR)/echo time (TE)/inversion time (TI) = 7040/2.612/900 ms, 0.9 mm isotropic resolution, field of view (FOV) = 230 × 230 mm, number of excitations (NEX) = 0.5, or a using a 3D magnetization prepared rapid gradient echo (sequence, TR/TE/TI = 1900/2.32/900 ms, 0.9 mm isotropic resolution, FOV = 230 × 230 mm, generalized auto-calibrating partial parallel acquisition, acceleration factor of 2 depending on site. Full acquisition details are documented elsewhere.^26^

Cortical surface reconstruction and segmentation was performed with FreeSurfer image analysis suite (version 6.0.0) (http://freesurfer.net/). The automized processing stream includes motion correction, removal of non-brain tissue, automated Talairach transformation, intensity correction, volumetric segmentation, cortical surface reconstruction and parcellation. Quality checks included skull stripping and pial surface errors, intensity normalization, white matter segmentation errors and were conducted on Freeview after processing and before statistical analysis. Entorhinal volume was derived from the Desikan-Killiany atlas.^37^ Hippocampal volumes were derived based on an atlas derived from combining high-resolution *ex vivo* data and *in vivo* data.

### Cognitive assessments

In Cohort 1, intact cognitive status was pre-determined. Details on the cognitive assessment are outlined elsewhere.^26^ Participants were assessed on the California Verbal Learning Task, the Montreal Cognitive Assessment and Digital Span to test for cognitive differences between *APOE* ε4 carriers and non-carriers.^27^^,^^38^ In Cohort 2, the Addenbrooke's cognitive evaluation and the Rey-Osterrieth complex figure test were available to confirm the sample was free of cognitive impairment and that there were no differences between *APOΕ* ε4 carrier and non-carriers. Spatial navigation performance was measured using the Virtual Supermarket Task adopted by the European Prevention of Alzheimer’s Dementia Consortium to assess the efficacy of potentially Alzheimer’s disease modifying treatments. Details for the spatial navigation task can be found elsewhere.^9^^,^^39^^,^^40^

### Statistical analysis

The data were analysed using RStudio (version 1.0.153). Linear regression models were specified with entorhinal and hippocampal volume as outcome variables, and DHA and *APOE* genotype as predictors (including an interaction term). Models were adjusted for age, sex, education, test centre and total estimated intracranial volume. Additional dietary variables such as total intake of green vegetables and fruit did not contribute to overall model fit based on the Bayesian information criterion criteria and were not retained in the final models. In the spatial navigation dataset, adjustments for test centre and intracranial volume were dropped as volumetric MRI was not the outcome variable and data collection took place at one site only. Standardized residuals were extracted and plotted against fitted values to examine underlining assumption of normal distribution and heteroscedasticity. In the case of significant *APOE* ×DHA interactions, *posthoc* linear models were specified with *APOΕ* ε4 carriers (ε3ε4) and non-carriers (ε3ε3) separately. All statistical tests are two-tailed: *P* < 0.05. Partial eta squared (*n*_p_^2^) was used as a measure of effect sizes and was derived from lmSupport package in R (https://cran.r-project.org/web/packages/lmSupport). *n*_p_^2^ is the ratio of variance associated with an effect plus that effect and its associated error variance (*n*_p_^2^ = SS_effect_/SS_effect_ + SS_error_).

### Data availability

The authors have carefully documented all data and materials used to conduct the research in this article and agree to share anonymized data by request from any qualified investigator.

## Results

The participant characteristics for cohorts 1 and 2 are summarized in Tables 1 and 2.

**Table 1:** Participant’s characteristics in Cohort 1

Characteristic				
	Total (*n* = 53)	*APOE genotype*		
		ε3ε4 carriers (*n* = 15)	ε3ε3 carriers (*n* = 38)	*P* value
Age (years)	64.2 (7.2)	65.0 (7.9)	64.0 (6.9)	0.65
Sex (male/female)	21/33	7/9	15/22	
Education (years)	14.2 (2.9)	14.5 (2.8)	14.1 (3.0)	0.63
Serum free DHA (µg/ml)	1.23 (0.63)	1.22 (0.59)	1.23 (0.66)	0.96
Serum LPC DHA (µg/ml)	2.15 (1.36)	2.23 (1.49)	2.10 (1.26)	0.43
Blood pressure				
Systolic (mm Hg)	133 (17)	121 (23)	126 (14)	0.61
Diastolic (mm Hg)	74 (7.6)	72 (7.3)	75 (7.8)	0.10
BMI (kg/m^2^)	26.9 (4.0)	27.1 (4.9)	26.9 (3.8)	0.87
Serum glucose (mmol/l)	5.19 (0.57)	5.26 (0.38)	5.17 (0.63)	0.63
Serum cholesterol (mmol/l)				
Total	5.08 (0.9)	5.04 (1.2)	5.12 (0.8)	0.82
HDL	1.42 (4.5)	1.39 (5.2)	1.44 (3.9)	0.81
Serum TG (mmol/l)	1.52 (0.5)	1.59 (0.6)	1.47 (0.4)	0.48
Serum BDNF (pg/ml)	18958 (4676)	19359 (4702)	18144 (4589)	0.13
Brain volume				
Hippocampal volume (ratio of total intracranial volume)	0.0045 (0.00038)	0.0044 (0.00046)	0.0045 (0.00035)	0.36
Entorhinal volume (ratio of total intracranial volume)	0.0024 (0.00031)	0.0023 (0.00025)	0.0024 (0.00033)	0.29
Cognition				
CVLT (delayed free recall)	10.99 (2.3)	10.43 (2.7)	11.16 (2.3)	0.28
MOCA (delayed recall)	3.11 (1.3)	3.07 (1.2)	3.24 (1.4)	0.68
MOCA (total)	27.87 (1.7)	27.73 (1.7)	27.93 (1.8)	0.72
Digital span (total score)	19.01 (3.4)	18.80 (3.2)	19.79 (3.6)	0.37

Data are presented as mean (SD) for normally distributed data or median for non-normal distributions. The two groups were compared by an independent sample *t*-test. Serum free DHA is measured as total DHA in serum, in the free/non-esterified fatty acid form. BDNF, brain derived neurotrophic factor; CVLT, California Verbal Learning Test, DHA, docosahexaenoic acid; HDL, high density lipoprotein, LPC; Lysophosphatidylcholine; MOCA, Montreal Cognitive Assessment; TG, triglycerides

**Table 2:** Participant characteristics in Cohort 2

Characteristic	Mean (SD)			
	Total (*n* = 46)	APOE genotype		
		ε3ε4 carriers (*n* = 22)	ε3ε3 carriers (*n* = 24)	*P* value
Socio-demographic				
Age (years)	61.30 (5.6)	60.82 (5.7)	61.75 (5.7)	0.58
Sex (male/female)	15/31	4/18	11/13	
Education (years)	14.4 (5.4)	14.5 (2.9)	14.4 (3.6)	0.72
Erythrocyte DHA (% of total FA)	2.64 (0.71)	2.76 (0.73)	2.52 (0.62)	0.25
Blood pressure				
Not medicated	36	18	18	0.61
Medicated	7	3	1	0.10
Cholesterol				
Not medicated	39	19	20	0.55
Medicated	4	2	2	0.81
Cognition				
ACE total score	94 (3.7)	93 (5.4)	94 (2.1)	0.55
ROCF				
Copy	32 (2.8)	32 (2.8)	32 (2.9)	0.55
Recall	19 (5.8)	17 (5.2)	20 (6.1)	0.08

Data are presented as mean (SD) for normally distributed data or median (IQR) for non-normal distributions. The two groups were compared by an independent sample *t*-test. ACE, Addenbrooke's Cognitive Examination; DHA, docosahexaenoic acid; FA, fatty acids; ROCF, Rey–Osterrieth complex figure

### Serum DHA associations with entorhinal and hippocampal volume

**Figure 1:**
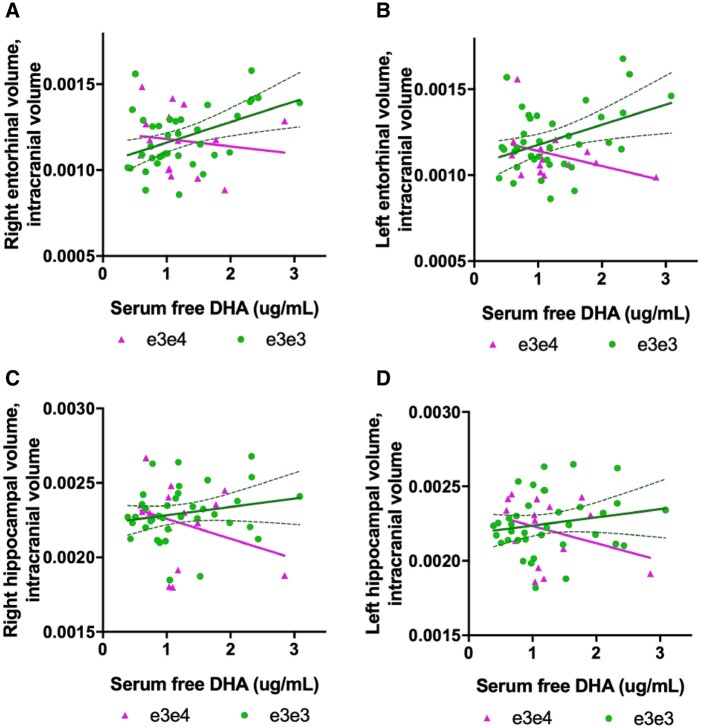
**Serum DHA associations with entorhinal and hippocampal brain volume from Cohort 1 (*n* = 53).** (**A, B**) There was a significant interaction between *APOE* genotype and DHA on left entorhinal volume (*t* = −2.20, *P* = 0.03). In ε3ε3 carriers (*n* = 38), serum-free DHA was significantly associated with right entorhinal volume and explained 20% of volume variability (*R*^2^ = 0.20, *P* = 0.005). Serum free DHA explained 8% of the variability in the left entorhinal volume (*R*^2^ = 0.08, *P* = 100). (**C, D**) No interaction between serum free DHA levels and *APOE* genotype on hippocampal volume was found, and no main effects of serum free DHA on hippocampal volume were found, although there was a trend towards significance. Confidence intervals represented by dotted curve lines are shown in for associations in the ε3ε3 groups.

Serum free DHA (in the free/non-esterified fatty acid form) predicted right (*t* = 2.15, *P* = 0.03, *n*_p_^2^ = 0.09) and left (*t* = 2.33, *P* = 0.02, *n*_p_^2^ = 0.11; Fig. 1A and B) entorhinal volume. There was a significant interaction between serum free DHA and *APOE* genotype status on the left entorhinal volume (*t* = −2.20, *P* = 0.03, *n*_p_^2^ = 0.10), with a trend evident for the right side (*t* = −2.00, *P* = 0.05, *n*_p_^2^ = 0.09). Independent linear models for *APOE* ε3ε3 and *APOE* ε3ε4 carrier groups revealed a positive association between serum free DHA levels and entorhinal volume in ε3ε3 carriers only (for both hemispheres: left *t* = 2.67, *P* = 0.01; right *t* = 2.28, *P* = 0.02). The DHA × *APOE* interaction was not significant for hippocampal volume, although there was a trend towards significance (right hemisphere: *t* = 1.72, *P* = 0.09; Fig. 1C and D).

### Serum LPC associations with entorhinal and hippocampal volume

LPC data were available from one of the two research sites in Cohort 1 (*n* = 30). LPC DHA predicted right hippocampal volume (*t* = 2.31, *P* = 0.03, *n*_p_^2^ = 0.22), with a significant LPC DHA × *APOE* interaction (*t* = −2.24, *P* = 0.03, *n*_p_^2^ = 0.19) and a positive and negative association trend evident in *APOE* ε3ε3 carriers and *APOE* ε3ε4 carrier’s respectively, which was not significant. Against predictions, there was no main effect of LPC DHA and entorhinal volume, suggesting the serum free DHA is associated with entorhinal cortex, but the LPC fraction is more strongly associated with the hippocampus in this sample.

### APOE effects on brain volume

We also investigated if the *APOE* genotype effects entorhinal cortex and hippocampus volume by removing the DHA predictor from the model which may have pulled variance from the *APOE* genotype. No main effects of *APOE* were found on entorhinal cortex volume (left: *t* = −1.71, *P* = 0.09; right: *t* = −0.47, *P* = 0.64) or hippocampus volume (left: *t* = −0.76, *P* = 0.44; right *t* = −1.44, *P* = 0.15), adjusting for age, sex, education, test site and total intracranial volume. See Supplementary Table 1 for a summary of the DHA effects on brain volume.

### DHA associations with spatial navigation

**Figure 2:**
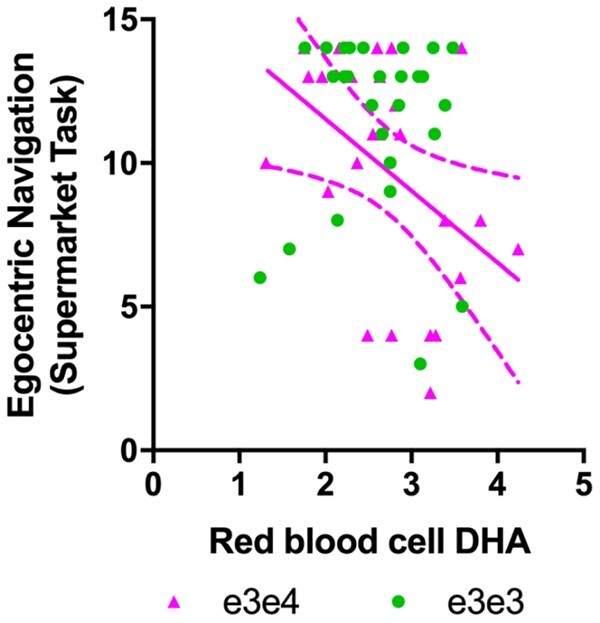
**DHA association with spatial navigation performance from Cohort 2 (*n* = 46).** There was a significant interaction between *APOE* genotype and DHA on left egocentric path integration (*t* = −2.06, *P* = 0.04). Total DHA in erythrocytes was inversely related to egocentric path integration in cognitively intact *APOE ε3ε4* carriers (*n* = 22) only. No significant association was found in the ε3ε3 carrier group.

In Cohort 2, we examined associations between erythrocyte DHA (the available blood DHA measure), and navigational processes vulnerable to early Alzheimer’s disease. Specifically, we tested DHA associations with boundary-based place memory and egocentric path integration (*n* = 46). Both processes tap into grid-cell mechanisms in the entorhinal cortex, which translate information to place cells in the hippocampus.^41^ There was a main effect of DHA on boundary-based place memory, which was marginally significant (*t* = −2.017, *P* = 0.058). The effect of DHA on egocentric path integration was not significant (*t* = 1.61, *P* = 0.12) although as expected, *APOE* genotype modulated DHA associations with egocentric path integration (*t* = −2.06, *P* = 0.04). DHA was inversely associated with path integration (*b* = −0.834, *t* = 2.69, *P* = 0.01) in ε3ε4, but not in ε3ε3 (*b* = 0.31, *t* = 1.48, *P* = 0.15; Fig. 2). These findings imply that higher circulating DHA predicts worse path integration performance in the ε4 carrier group only. See Supplementary Table 1 for a summary of effects on spatial navigation.

### DHA associations with brain regions beyond the medial temporal lobe

Finally, we investigated if the *APOE* genotype and DHA interact to predict volumes of other Alzheimer’s disease vulnerable brain regions in the human spatial navigation network, namely the precuneus and posterior cingulate cortex. No significant interactions (or main effects of serum free DHA or LPC DHA) on brain volume were found (Supplementary Table 2), suggesting that the effects of DHA observed here are concentrated in the entorhinal cortex and hippocampus.

## Discussion

Our findings imply that the *APOE* ε4 allele alters associations between circulating DHA and volumes of the entorhinal cortex and hippocampus in adults without dementia, almost a decade before the expected age of Alzheimer’s disease onset. Circulating serum DHA predicted greater entorhinal cortex volume, with a significant interaction between DHA and APOE genotype. As predicted, the positive association between DHA and entorhinal volume was evident in non *APOE* ε4 carriers only. Our results also show that although there was no main effect of serum DHA on egocentric path integration, there was a significant interaction between DHA and APOE, with serum DHA being inversely correlated with path integration in *APOE* ε4 carriers only. We can speculate that impaired blood−brain barrier function and reduced DHA transport to the entorhinal cortex and hippocampus are plausible mechanisms behind this inverse association, which warrant further investigation. Unexpectedly, serum DHA was not positively associated with path integration in non *APOE* ε4 carriers. Together, the results imply that disrupted DHA absorption from the blood to the brain may exist in the genetically at risk of Alzheimer’s disease adult population, and may mediate the presentation of Alzheimer’s disease markers.

Serum DHA predicted greater entorhinal volume in non-demented older adults, consistent with previous findings for a beneficial influence of circulating DHA (from serum or erythrocytes) on brain health.^5^^,^^7^^,^^21^^,^^42^^,^^43^ The entorhinal cortex has one of the highest concentrations of lipoprotein receptors in the brain (due to the presence of APOE receptors LRP1) which are involved in DHA tissue delivery to neurons and the clearance of amyloid β.^57^ This may explain why DHA was associated with both these regions and not the precuneus cortex or the posterior cingulate cortex. However, the beneficial association of DHA with brain volume was exclusive to non-ε4 carriers in our study, consistent with two similar observational studies.^44^^,^^45^ Daiello et al.^46^ previously showed that DHA supplementation predicted the preservation of the cerebral cortex grey matter and the hippocampus in non-ε4 carriers only, pointing to a neuroprotective effect of DHA that is at least partially exclusive to adults who do not bear the risk of the ε4 allele.

The spatial navigation study from Cohort 2 supported this theory. Among adults ε4 allele carriers, circulating DHA predicted worse navigation proficiency. To the best of our knowledge, this is the first report of a significant association between circulating DHA and Alzheimer’s disease vulnerable spatial navigation performance. Path integration, a sub-process involved in navigation ability, involves the capacity to use self-motion cues (or movements cues) to update and learn spatial location information in relation to a start location^47^ and is particularly vulnerable to early Alzheimer’s disease pathophysiology.^48^ This process relies crucially on the structural integrity of the entorhinal cortex and hippocampus that were notably associated with serum DHA here.^49^^,^^50^ Almost a decade ago, He et al.,^6^ demonstrated that increased brain DHA (via supplementation) significantly increased the number of proliferating hippocampal cells and subsequently improved spatial learning performance in rats on the Morris Water Maze. In our *APOE* ε4 group, increased circulating DHA was associated with decreased navigation performance, supporting evidence that *APOE* ε4 disrupts blood−brain barrier function predicting cognitive decline.^20^^,^^24^^,^^51^

A landmark paper by Montague and colleagues provides important insights into a deficit blood−brain barrier transport system in *APOΕ* ε4 carriers. The authors report that *APOE* ε4 carriers present with blood–brain barrier breakdown in the hippocampus and medial temporal lobes leading to cognitive decline. Lower brain uptake of DHA in older *APOE* ε4 mice has also been shown to limit the accumulation of DHA in cerebral tissues, providing a potential mechanistic explanation for the inverse association between DHA and spatial navigation in *APOE* ε4 shown here.^25^ Other explanations for APOE related changes in DHA metabolism beside blood−brain barrier function include (i) ε4 carrier status results in physiological dysregulation that is associated with both lower brain DHA uptake (and resultant higher blood levels) and deleterious changes to medial temporal lobe physiology and function, or (ii) there is greater DHA uptake in adipose tissue for storage in *APOE* ε4 carriers with less available for brain tissue absorption via the blood−brain barrier. Other potential explanations for the increase in free serum DHA among *APOE* ε4 may be that this reflects greater activation of phospholipase A2, which liberates esterified DHA from phospholipid,^52^ suggesting that the increase in DHA is a biomarker of another processes such as enhanced vascular inflammation as opposed to being directly linked to Alzheimer’s disease pathology. All mechanisms warrant further investigation.

We examined the effect of *APOE* ε4 on the association between circulating DHA, entorhinal cortex, hippocampal volume and spatial navigation, which is uncommon as most studies focus on memory or other cognitive functions. Strengths of our study include a rigorous DHA analysis, including the serum LPC−DHA lipid fraction, a comprehensive phenotyping of participants, adjusting for confounders, as well as the inclusion of a virtual reality spatial navigation diagnostic test of Alzheimer’s disease. The findings produced in this study have the following limitations however, (i) although a unified model with an interaction term is the optimum method to test effect modification, an important limitation is that more statistical power is required than for association testing, and thus false-negative results may be seen in smaller samples. There were fewer ε4 carriers in Cohort 1, compared with Cohort 2, which may account for why in in *APOE* ε4 carriers we found a significant inverse association between DHA and navigation performance, but a null association between DHA and entorhinal−hippocampal brain volume. (ii) Likewise, the moderate sample sizes do not preclude the possibility that our findings could be observed by chance. These results will nevertheless play an important role in hypothesis-generating for future cross-sectional studies and RCTs. (iii) While spatial navigation crucially relies on the integrity and function of the entorhinal cortex and the hippocampus, we cannot directly relate the participants across Cohort 1 and 2. Future studies should thus examine if entorhinal and hippocampal brain volume directly mediates the relationships between DHA and spatial navigation. (iv) Given the observational nature of the study, and that DHA (fish intake) is a component of an overall healthy diet,^53^ we cannot rule out the possibility of confounding residual and that the DHA-brain phenotype associations are attributable to other dietary factors. To address this potential confound, we tested various other dietary factors such as vegetable and fruit intake, which did not contribute to a significant amount of variance in the outcome variables of interest and therefore, where not retained in the downstream analysis. Given that brain and serum DHA has been previously linked to a range of neuro-protective processes in animal and human models, it is unlikely that the association are due to other diet derived bioactives.

In conclusion, we provide novel preliminarily evidence that the *APOE* genotype modifies DHA associations with brain volume and spatial navigation ability, typically affected in the first stages of Alzheimer’s disease. Future studies should examine the mechanisms behind the *APOE* genotype modulating effect of DHA, brain volume and cognitive function associations, particularly blood−brain barrier integrity. Future positron emission tomography studies needed to measure rates of DHA incorporation from serum into the brain,^51^ which would confirm if DHA uptake to the brain is reduced in older *APOE* ε4 carriers, leading to spatial navigation impairment. As over 50% of *APOE* ε4 carriers do not develop clinical Alzheimer’s disease, longitudinal studies are clearly required to determine whether *APOE ε4,* coupled with disrupted DHA absorption to the brain, has diagnostic utility, and can predict conversion to clinical Alzheimer’s disease with a high degree of accuracy.^54^ Another important line of research will be to examine a therapeutic target in *APOE* ε4 carriers to mitigate the negative effect of the allele on brain health. For example, it is possible that cyclophilin A inhibitors might suppress the pathway that is believed to cause blood−brain barrier breakdown in the cerebral blood vessels of *APOE* ε4 carriers, and thereby may slow spatial navigation impairment and other cognitive functions that rely on blood supply to the brain.^24^^,^^55^ Moreover, supplementation trials may need to focus on higher doses of DHA to ensure adequate brain delivery in *APOE* ε4 carriers, as previously suggested.^56^ Finally, understanding whether different blood lipid fractions differentially supply DHA to the entorhinal cortex and hippocampus in humans may refine DHA intervention approaches in dietary treatment trials for Alzheimer’s disease.

## Supplementary material

Supplementary material is available at *Brain Communications* online.

## Supplementary Material

fcab085_Supplementary_Data

## Abbreviations

ACE =: Addenbrookes cognitive examination
APOE =: apolipoprotein E
BDNF =: brain-derived neurotrophic factor
BMI =: body mass index
CI =: confidence interval
CVLT =: California verbal learning test
DHA =: docosahexaenoic acid
FAs =: fatty acids
GLM =: general linear model
HDL =: high-density lipoprotein
LPC =: lysophosphatidylcholine
MOCA =: Montreal Cognitive Assessment
ROCF =: Rey–Osterrieth Complex Figure
TG =: triglyceride
TIV =: total intracranial volume
ω-3 PUFA =: omega-three polyunsaturated fatty acids
*n*_p_^2^ =: partial eta squared
