# Supplementary material for: APOE ε4 alters associations between docosahexaenoic acid and preclinical markers of Alzheimer’s disease
Source: Brain Commun. 2021 May 11;3(2):fcab085. doi: 10.1093/braincomms/fcab085 (PMC8112902; doi:10.1093/braincomms/fcab085)
Supplement: fcab085_Supplementary_Data [file fcab085_supplementary_data.docx]

***Supplementary Table 1.*** *Summary of statistical effects of APOE and DHA on relative entorhinal cortex volumes, hippocampal volume and spatial navigation (n=46)*.

|  | APOE | | Serum DHA | | APOE x DHA interaction | |
| --- | --- | --- | --- | --- | --- | --- |
| **Relative brain volume** | t value | P value | t value | P value | t value | P value |
| Left Hippocampus | 0.954 | 0.34 | 1.444 | 0.156 | -1.419 | 0.16 |
| Right Hippocampus | 0.797 | 0.42 | 1.721 | 0.09* | -1.718 | 0.09 |
| Left Entorhinal | 1.229 | 0.22 | **2.328** | **0.02** | **-2.208** | **0.03** |
| Right Entorhinal | 1.620 | 0.11 | **2.153** | **0.03** | **-2.000** | **0.05** |
|  | APOE | | Serum LPC-DHA | | APOE x DHA interaction | |
|  | t value | P value | t value | P value | t value | P value |
| Left Hippocampus | 1.234 | 0.23 | 1.610 | 0.122 | -1.631 | 0.11 |
| Right Hippocampus | 1.957 | 0.06 | **2.310** | **0.03** | **-2.236** | **0.03** |
| Left Entorhinal | 1.706 | 0.09 | 0.691 | 0.493 | 0.659 | 0.51 |
| Right Entorhinal | 0.331 | 0.74 | 0.989 | 0.32 | -1.008 | 0.31 |
|  | APOE | | Erythrocyte DHA | | APOE x DHA interaction | |
| **Spatial navigation** | t value | P value | t value | P value | t value | P value |
| Egocentric Path Integration | 1.234 | 0.23 | 1.610 | 0.12 | **-2.056** | **0.04** |
| Boundary-based Place Memory | 0.234 | 0.01 | **-2.017** | **0.058** | 1.143 | 0.26 |

Abbreviations: APOE = Apolipoprotein E; LPC DHA= serum lysophosphatidylcholine DHA; x=interaction

***Supplementary Table 2.*** *The effect of serum DHA and LPC DHA on precuneus and posterior cingulate cortex volume.*

| **Right precuneus** | T value | P value |
| --- | --- | --- |
| Education | 0.882 | 0.38 |
| TIV | 4.330 | 0.00*** |
| Age | -2.403 | 0.02* |
| Sex | 1.279 | 0.21 |
| Centre | 1.913 | 0.06 |
| APOE | -0.734 | 0.46 |
| DHA | 0.064 | 0.94 |
| APOE*DHA | -0.045 | 0.96 |
| *LPC-DHA Model* |  |  |
| LPC-DHA | 1.573 | 0.12 |
| LPC-DHA*APOE | 1.453 | 0.15 |
| **Left precuneus** | T value | P value |
| Education | -0.139 | 0.88 |
| TIV | 4.257 | 0.00*** |
| Age | -0.641 | 0.52 |
| Sex | 1.355 | 0.18 |
| Centre | .266 | 0.03* |
| APOE | -0.382 | 0.70 |
| DHA | -0.322 | 0.74 |
| APOE*DHA | 0.258 | 0.79 |
| *LPC-DHA Model* |  |  |
| LPC-DHA | 0.453 | 0.65 |
| LPC-DHA*APOE | 0.322 | 0.74 |
| **Right posterior cingulate** | T value | P value |
| Education | -0.471 | 0.63 |
| TIV | 3.224 | 0.00*** |
| Age | -1.253 | 0.217 |
| Sex | -0.853 | 0.39 |
| Centre | 2.611 | 0.01* |
| APOE | 0.060 | 0.95 |
| DHA | -0.315 | 0.75 |
| APOE*DHA | 0.280 | 0.78 |
| *LPC-DHA Model* |  |  |
| LPC-DHA | 1.169 | 0.24 |
| LPC-DHA*APOE | 1.068 | 0.29 |
| **Left posterior cingulate** | T value | P value |
| Education | -0.431 | 0.66 |
| TIV | 3.865 | 0.00*** |
| Age | -2.505 | 0.02* |
| Sex | 0.963 | 0.34 |
| Centre | 1.701 | 0.09 |
| APOE | 0.146 | 0.88 |
| DHA | 0.502 | 0.62 |
| APOE*DHA | -0.336 | 0.73 |
| *LPC-DHA Model* |  |  |
| LPC-DHA | 1.094 | 0.28 |
| LPC-DHA*APOE | 1.009 | 0.31 |

Linear models were fitted to cohort one. Model predictors are listed in the left column. LPC-DHA and total serum DHA (i.e. DHA) were specified in separate linear models. All other predictors remained the same in the DHA and LPC-DHA models.

Significance codes: ***=0.001**=0.01 *=0.05

Abbreviations: APOE: Apolipoprotein; TIV: Total intracranial volume; LPC DHA= serum lysophosphatidylcholine DHA; x=interaction
